# Difference Between Day and Night Temperatures Affects Stem Elongation in Tomato (*Solanum lycopersicum*) Seedlings via Regulation of Gibberellin and Auxin Synthesis

**DOI:** 10.3389/fpls.2020.577235

**Published:** 2020-12-08

**Authors:** Kinuka Ohtaka, Akiko Yoshida, Yusuke Kakei, Kosuke Fukui, Mikiko Kojima, Yumiko Takebayashi, Kanako Yano, Shunsuke Imanishi, Hitoshi Sakakibara

**Affiliations:** ^1^RIKEN Center for Sustainable Resource Science, Yokohama, Japan; ^2^Graduate School of Bioagricultural Sciences, Nagoya University, Nagoya, Japan; ^3^Department of Chemical and Biological Sciences, Faculty of Science, Japan Women’s University, Tokyo, Japan; ^4^Department of International Environmental and Agricultural Science, Tokyo University of Agriculture and Technology, Tokyo, Japan; ^5^NARO, Institute of Vegetable and Floriculture Science, Tsu, Japan; ^6^Department of Biochemistry, Okayama University of Science, Okayama, Japan

**Keywords:** auxin, DIF, gibberellin, *Solanum lycopersicum*, stem elongation, tomato

## Abstract

Temperature is a critical environmental factor governing plant growth and development. The difference between day temperature (DT) and night temperature (NT), abbreviated as DIF, influences plant architecture. Subjecting plants to artificial DIF treatments is an effective strategy in ornamental horticulture. For example, negative DIF (when DT – NT < 0) generally inhibits stem elongation, resulting in dwarf plants. However, the mechanisms underlying stem growth regulation by DIF remains to be completely elucidated. In this study, we aimed to analyze the growth, transcriptome, and phytohormone profiles of tomato (*Solanum lycopersicum*) seedlings grown under different DIF treatments. Under positive DIF (when DT – NT > 0), in contrast to the control temperature (25°C/20°C, DT/NT), high temperature (30°C/25°C) increased stem length and thickness, as well as the number of xylem vessels. Conversely, compared with the positive high temperature DIF treatment (30°C/25°C), under negative DIF treatment (25°C/30°C) stem elongation was inhibited, but stem thickness and the number of xylem vessels were not affected. The negative DIF treatment decreased the expression of gibberellin (GA)-, auxin-, and cell wall-related genes in the epicotyl, as well as the concentrations of GAs and indole-3-acetic acid (IAA). The expression of these genes and concentrations of these hormones increased under high temperature compared to those under the control temperature positive DIF. Our results suggest that stem length in tomato seedlings is controlled by changes in GA and IAA biosynthesis in response to varying day and night temperatures.

## Introduction

Plants modulate their growth according to their environmental conditions. In particular, temperature is a critical factor affecting plant growth. Each plant species has a suitable temperature range. Within this range, higher temperatures generally promote shoot growth, including leaf expansion and stem elongation and thickening. However, temperatures above the optimal range suppress growth. In addition to the absolute temperature, the difference in temperature between day and night can affect growth. Normally, night temperatures are lower than daytime temperatures, and plants modulate their growth pattern and metabolism in response to this temperature change. Altering the temperature difference between day and night is one method through which plant growth is controlled in ornamental horticulture. The difference between day temperature (DT) and night temperature (NT), abbreviated as DIF, is defined as DT– NT. Positive DIF occurs when DT is higher than NT, zero DIF occurs when DT is the same as NT, and negative DIF occurs when DT is lower than NT. Negative DIF can be used to control plant height as an alternative to agricultural chemicals (Shimizu, 2007). While the effects were plant species-dependent, it has been reported that negative DIF inhibits stem elongation compared to positive DIF in various plants, including *Lilium longiflorum*, *Dendranthema grandiflora*, *Cucumis sativus* (Shimizu, 2007), and *Solanum lycopersicum* (Went, 1944; de Koning, 1988).

Previous studies have suggested that phytohormones are involved in regulating plant growth in response to temperature. Gibberellin (GA) is known to play a key role in stem elongation (Davière and Achard, 2013; Binenbaum et al., 2018; Ferrero et al., 2019). Indole-3-acetic acid (IAA), an auxin, also plays an important role in cell elongation in the hypocotyl, the epicotyl, and other organs (Leyser, 2018; Zhao, 2018). In *Arabidopsis thaliana*, higher temperatures promote hypocotyl elongation mediated by phytochrome-interacting factor 4 (PIF4)-dependent auxin biosynthesis (Franklin et al., 2011; Nomoto et al., 2012; Sun et al., 2012). It has been demonstrated that PIF4 function is regulated by GA via DELLA proteins, which are key negative regulators of GA signaling (Koini et al., 2009; Stavang et al., 2009).

Studies have found that stem elongation under different DIF treatments is accompanied by a change in GA content in *Campanula isophylla* and *Pisum sativum* (Jensen et al., 1996; Grindal et al., 1998; Stavang et al., 2005). In *P. sativum*, inhibition of stem elongation under negative DIF was weaker in GA-related mutants than that in the wild type (Grindal et al., 1998). In *A. thaliana*, non-bioactive GA_29_ content was lower under a negative DIF treatment than that under a positive DIF treatment, while IAA concentration was higher under a positive DIF treatment than that under a negative DIF treatment (Thingnaes et al., 2003). In *Raphaus sativus* L., differences in stem elongation under DIF treatments followed a similar pattern to changes in IAA content (Hayata et al., 2001). These studies suggest the involvement of these hormones in the effect of DIF on stem elongation. However, these hormones and the expression of their genes have not been investigated in detail. Further, temperature not only affects stem elongation but also stem thickness; however, the effect of DIF on vascular development has not been properly characterized to date.

In this study, we aimed to elucidate the mechanisms underlying stem growth regulation by DIF. To this end, we examined the effects of different DIF treatments on tomato (*S. lycopersicum*), which is one of the most important vegetable crops. Our analyses of growth, transcriptomes, and hormones strongly suggest that negative DIF-dependent inhibition of stem elongation is mediated by the repression of GA and IAA synthesis accompanied by the regulation of cell wall-related genes. We also report that negative DIF treatment has a minimal effect on stem thickening in tomato seedling.

## Results

### Higher Temperatures Under Positive DIF Promote Stem Elongation and Thickening in Tomato Seedlings

**FIGURE 1:**
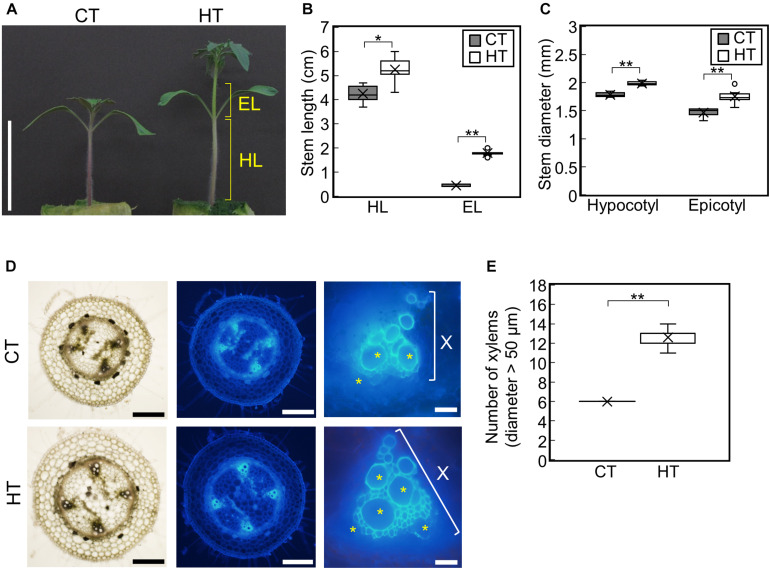
Effect of temperature on growth of tomato seedlings. Young tomato seedlings were grown for 7 days under 25°C/20°C (control temperature) and 30°C/25°C (high temperature). **(A)**, Growth of seedlings. Tomato seedlings were photographed at 7 days. Scale bar = 5 cm. **(B)**, Hypocotyl and epicotyl length. **(C)**, Hypocotyl and epicotyl diameter. Data are shown as boxplots (*n* = 7). **(D)**, Cross sections of hypocotyls. Asterisks indicate xylems (diameter > 50 μm). Scale bars = 500 μm (left and middle panels) and 50 μm (right panels). **(E)**, Number of xylems (diameter > 50 μm) in the cross section. Data are shown as boxplots (*n* = 5). Cross marks in boxplot indicate the mean values. *P < 0.01; **P < 0.001 (Student’s *t* test). CT, control temperature; HT, high temperature; HL, hypocotyl length; EL, epicotyl length; X, xylem area.

To examine the effect of temperature under positive DIF on plant growth, tomato seedlings (Managua RZ) were grown under control (25°C/20°C, CT) and high temperatures (30°C/25°C, HT) (Supplementary Figures 1A,B). The stem, hypocotyl, and epicotyl lengths and diameters were significantly greater under HT than those under CT (Figures 1A–C). Histological analysis of hypocotyl cross sections showed that the number of xylem vessels (diameter > 50 μm) were significantly greater under HT than that under CT (Figures 1D,E), indicating that higher temperatures under positive DIF promote stem elongation, stem thickening, and vascular development in tomato seedlings.

### Negative DIF Inhibits Stem Elongation, but Maintains Promotion of Stem Thickening

**FIGURE 2:**
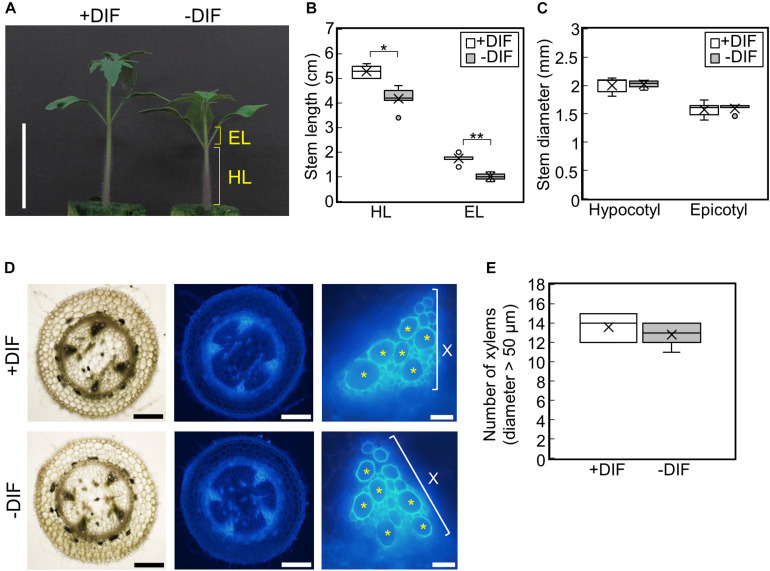
Effect of DIF treatment on growth of tomato seedlings. Young tomato seedlings were grown for 7 days under 30°C/25°C (positive DIF) and 25°C/30°C (negative DIF). **(A)**, Growth of seedlings. Seedlings of tomato were photographed at 7 days. Scale bar = 5 cm. **(B)**, Hypocotyl and epicotyl length. **(C)**, Hypocotyl and epicotyl diameter. Data are shown as boxplots (*n* = 5). **(D)**, Cross sections of hypocotyls. Asterisks indicate xylems (diameter > 50 μm). Scale bars = 500 μm (left and middle panels) and 50 μm (right panels). **(E)**, Number of xylems (diameter > 50 μm) in the cross section. Data are shown as boxplots (*n* = 5). Cross marks in boxplot indicate the mean values. *P < 0.01; **P < 0.001 (Student’s *t* test). +DIF, positive DIF; -DIF, negative DIF; HL, hypocotyl length; EL, epicotyl length; X, xylem area.

To examine the effect of negative DIF on tomato seedling growth, tomato seedlings were grown under HT (30°C/25°C, positive DIF) or with night and day temperatures reversed (25°C/30°C, negative DIF) (Supplementary Figures 1B,C). Under the negative DIF treatment, temperature-dependent stem elongation was inhibited both in the hypocotyl and epicotyl (Figures 2A,B). The difference in hypocotyl and epicotyl elongation between DIF treatments could be detected from 3 d after the onset of the treatments, and it became significant at 5 d (Supplementary Figures 2A–C). Conversely, hypocotyl and epicotyl thickness were similar under both DIF treatments (Figure 2C and Supplementary Figures 2D,E). The number of xylem vessels (diameter > 50 μm) was also similar (Figures 2D,E), indicating that the negative DIF treatment inhibited stem elongation without any negative effect on stem thickening. The size of cotyledons and true leaves was also similar under both treatments (Supplementary Figure 2F).

To determine whether these responses were cultivar-specific, we tested the effect of the same DIF treatments on four other tomato cultivars. Similar to Managua RZ, the negative DIF treatment decreased stem length in all four tomato cultivars, but it did not affect stem thickness (Supplementary Figure 3). This suggests that the growth responses observed are a common feature among tomato seedlings.

### Negative DIF Affects Gene Expression

**FIGURE 3:**
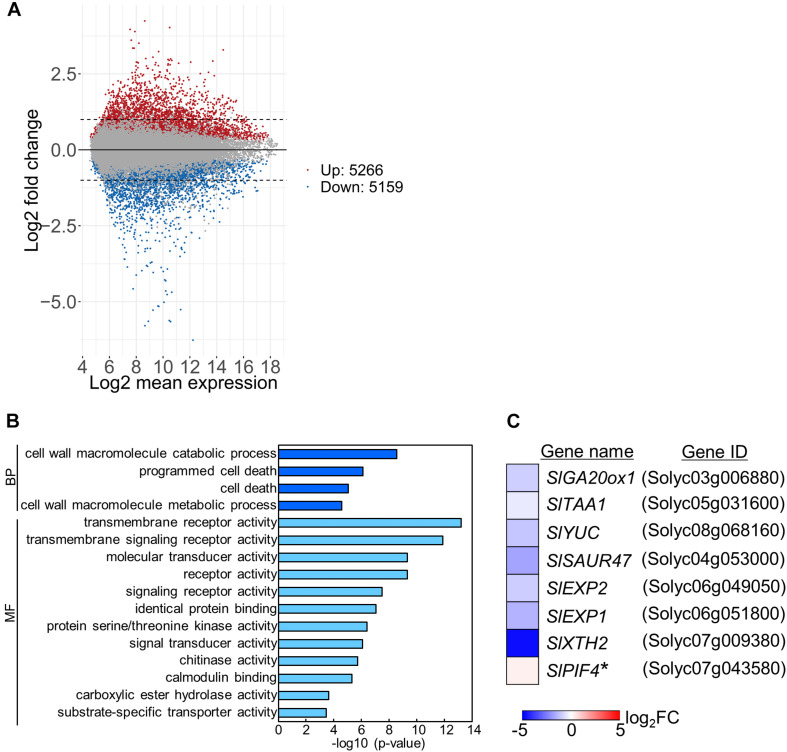
Transcriptome analysis of tomato under positive and negative DIF treatments. Total RNA was extracted from epicotyls of tomato seedlings grown for 7 days under positive DIF and negative DIF and subjected to microarray analysis with three biological replicates. **(A)**, MA plots of microarray data in the positive DIF and negative DIF treatments. Red and blue dots represent genes upregulated (Up: 5266 genes) and downregulated (Down: 5159), respectively, under negative DIF in comparison with positive DIF. Gray dots represent genes that did not significantly differ between DIF treatments. **(B)**, GO terms enriched among the DEGs (top 300 upregulated and top 300 downregulated genes; *P* < 0.05) identified. **(C)**, List of 8 genes associated with stem elongation. The relative expression level of each gene under negative DIF compared with that under positive DIF is shown on the heatmap, which represents the log_2_ fold-change (FC). The color scale is shown at the bottom. +DIF, positive DIF; -DIF, negative DIF; BP, Biological Process; MF, Molecular Function.

To examine the mechanisms underlying stem growth regulation under the DIF treatments, we analyzed epicotyl transcriptomes in seedlings grown under positive or negative DIF for 7 d (Supplementary Figures 1B,C) and explored differentially expressed genes (DEGs). Microarray analysis revealed more than 5000 DEGs, with some upregulated and downregulated by negative DIF treatment (Figure 3A). We performed gene ontology (GO) analysis using the top 300 upregulated and downregulated genes (*P* < 0.05) (Supplementary Tables 1, 2). Enriched GO terms in biological process were found in cell wall macromolecule catabolic/metabolic process and (programmed) cell death (Figure 3B). Since stem elongation is related to cell wall modification, we focused on cell wall-related genes for further analyses. In addition, hormone-related genes could be involved in temperature-dependent regulation of epicotyl elongation; consequently, GA and IAA-related genes were also included in further analyses. In total, seven DEGs associated with the cell wall, GA, and IAA, as well as a *PIF4* homolog possibly related to temperature-dependent hypocotyl elongation, were chosen for further analyses (Figure 3C).

### GA and IAA Biosynthesis Genes Were Downregulated in Stems Under the Negative DIF Treatment

**FIGURE 4:**
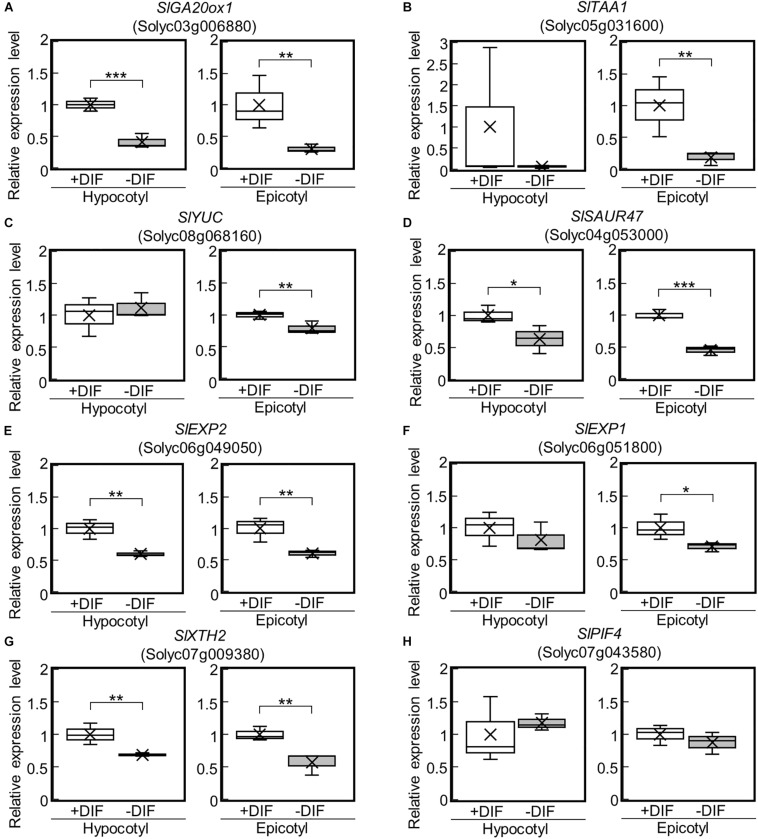
Expression of GA-, IAA-, and cell wall-related genes in epicotyls and hypocotyls under negative and positive DIF treatments. The expression of *SlGA20ox1* **(A)**, IAA-related genes (*SlTAA1*, *SlYUC*, *SlSAUR47*) **(B–D)**, cell wall-related genes (*SlEXP2*, *SlEXP1*, *SlXTH2*) **(E–G)**, and *SlPIF4* **(H)** in epicotyls and hypocotyls grown under positive and negative DIF treatments were analyzed using reverse transcriptase real-time PCR. Data are shown as boxplots (*n* = 3). Cross marks in boxplot indicate the mean values. *P < 0.1; **P < 0.05; ***P < 0.01 (Student’s *t* test). +DIF, positive DIF; -DIF, negative DIF.

Among the identified DEGs, a key gene of *de novo* GA biosynthesis, namely *GA20-oxidase* (*SlGA20ox1*: Solyc03g006880), was downregulated (Figure 3C). Using reverse transcriptase real-time PCR, we confirmed that the expression of *SlGA20ox1* was significantly downregulated in both hypocotyls and epicotyls under the negative DIF treatment (Figure 4A). The following genes involved in IAA biosynthesis were also among the downregulated DEGs: *YUCCA (YUC)*, which encodes a flavin monooxygenase-like enzyme; *TRYPTOPHAN AMINOTRANSFERASE OF ARABIDOPSIS (TAA)*; and IAA responsive factor *SMALL AUXIN UP RNA (SAUR)* (Figure 3C). Our reverse transcriptase real-time PCR analysis confirmed that *SlTAA1* (Solyc05g031600) and *SlYUC* (Solyc08g068160) were downregulated in tomato epicotyl tissues under the negative DIF treatment (Figures 4B,C). *SlSAUR47* (Solyc04g053000) was significantly downregulated in both hypocotyl and epicotyl tissues under the negative DIF treatment (Figure 4D).

### Cell Wall Modification Genes Were Downregulated in Stems Under the Negative DIF Treatment

In our transcriptome analysis, *expansins* (*SlEXP2*; Solyc06g049050 and *SlEXP1*; Solyc06g051800) and *Xyloglucan endotransglucosylase/hydrolase* (*SlXTH2*; Solyc07g009380) were downregulated (Figure 3C). Our reverse transcriptase real-time PCR analysis revealed that *SlEXP2* and *SlXTH2* were significantly downregulated in both hypocotyls and epicotyls under the negative DIF treatment (Figures 4E,G). *SlEXP1* was also downregulated in epicotyls under the negative DIF treatment (Figure 4F). These results are in line with the negative DIF treatment-driven repression of stem elongation.

We further examined the expression of *PIF4*, but the expression of *SlPIF4* (Solyc07g043580), a homolog of *A. thaliana PIF4*, was not affected by DIF treatment (Figure 4H).

### Higher Temperatures Under Positive DIF Upregulate Genes for GA and IAA Biosynthesis, as Well as Cell Wall Modification in Epicotyls

**FIGURE 5:**
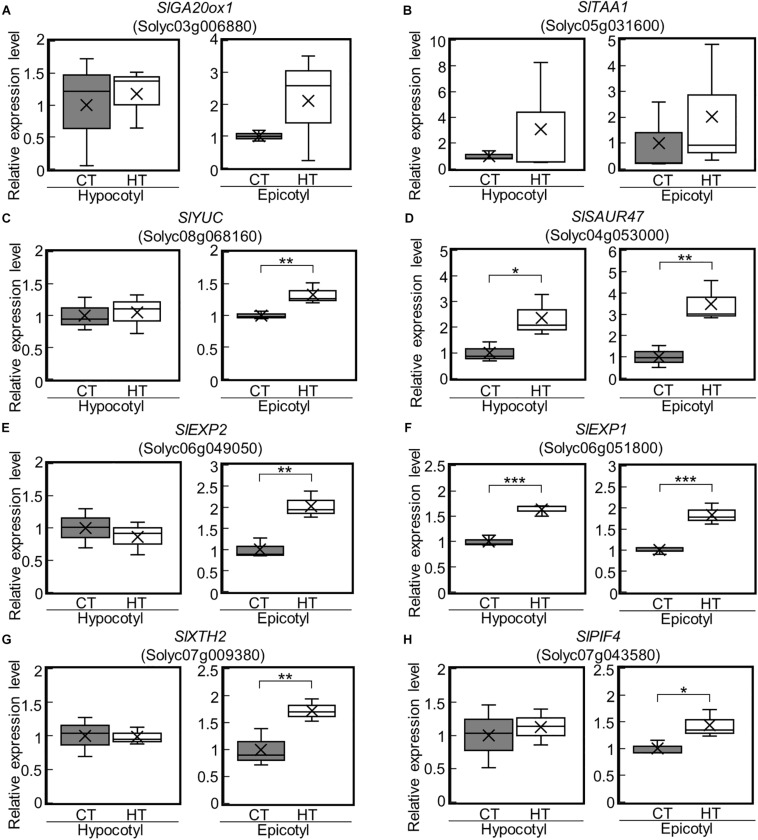
Expression of GA-, IAA-, and cell wall-related genes in epicotyls and hypocotyls under two different temperatures. The expression of *SlGA20ox1* **(A)**, IAA-related genes (*SlTAA1*, *SlYUC*, *SlSAUR47*) **(B–D)**, cell wall-related genes (*SlEXP2*, *SlEXP1*, *SlXTH2*) **(E–G)**, and *SlPIF4* **(H)** in epicotyls and hypocotyls grown under control and high temperatures were analyzed by reverse transcriptase real-time PCR. Data are shown as boxplots (*n* = 3). Cross marks in boxplot indicate the mean values. *P < 0.1; **P < 0.05; ***P < 0.01 (Student’s *t* test). CT, control temperature; HT, high temperature.

We next examined the expression of the DEGs under the CT (25°C/20°C) and HT (30°C/25°C) positive DIF treatments (Supplementary Figures 1A,B) using reverse transcriptase real-time PCR. *SlGA20ox1, SlYUC*, *SlEXP2*, and *SlXTH2* were upregulated in epicotyls under the HT treatment (Figures 5A,C,E,G). *SlSAUR47* and *SlEXP1* were also upregulated in both hypocotyls and epicotyls under the HT treatment (Figures 5D,F). These results are consistent with the positive DIF treatment-driven promotion of stem elongation. In addition, *SlPIF4* was upregulated in epicotyls under the HT treatment (Figure 5H).

### Phytohormone Concentrations Are Consistent With Stem Elongation and Gene Expression Patterns in Response to DIF Treatments

**FIGURE 6:**
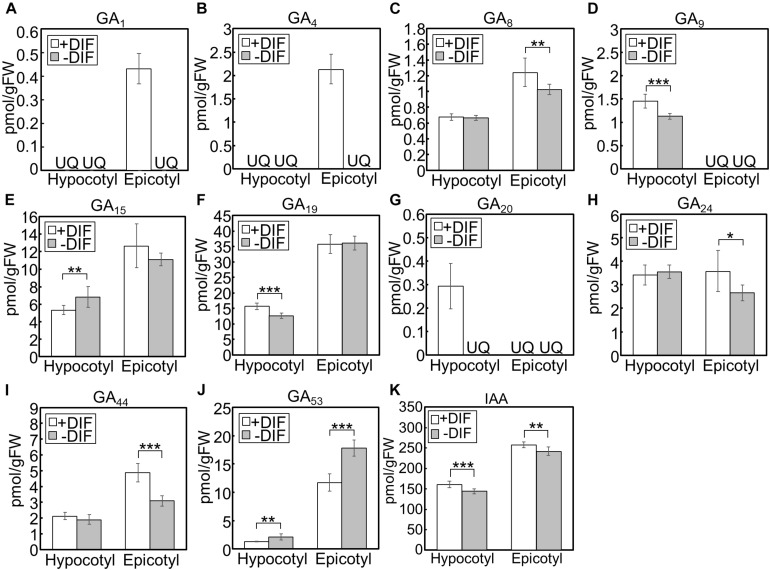
GA and IAA concentration in hypocotyls and epicotyls under negative and positive DIF treatments. Hypocotyls and epicotyls of tomato seedlings grown for 7 days under positive and negative DIF treatments were harvested and subjected to hormone analysis. The concentrations of GAs **(A–J)** and IAA **(K)** were quantified using liquid chromatography-tandem mass spectrometry. Data are represented as mean ± SD (*n* = 4 or 5). *P < 0.1; **P < 0.05; ***P < 0.01 (Student’s *t* test). GA_7_ and GA_12_ were below the quantification limit. +DIF, positive DIF; -DIF, negative DIF; FW, fresh weight; UQ, under quantification limit.

We further investigated the effect of growth temperature on phytohormone concentrations in hypocotyls and epicotyls (Figure 6). Under positive and negative DIF treatments, GA_1_ and GA_4_, bioactive forms, could only be quantified in epicotyls under the positive DIF treatment, whereas they were below the quantification limit in all other tissues (Figures 6A,B). Another bioactive form, GA_7_, was also below the quantification limit (Figure 6C). The concentrations of the precursors, including GA_9_, GA_19_, GA_20_, GA_24_, and GA_44_, were lower in hypocotyls or epicotyls, while that of GA_53_ was higher, under the negative DIF treatment compared with those under the positive DIF treatment (Figures 6D,F–J).

**FIGURE 7:**
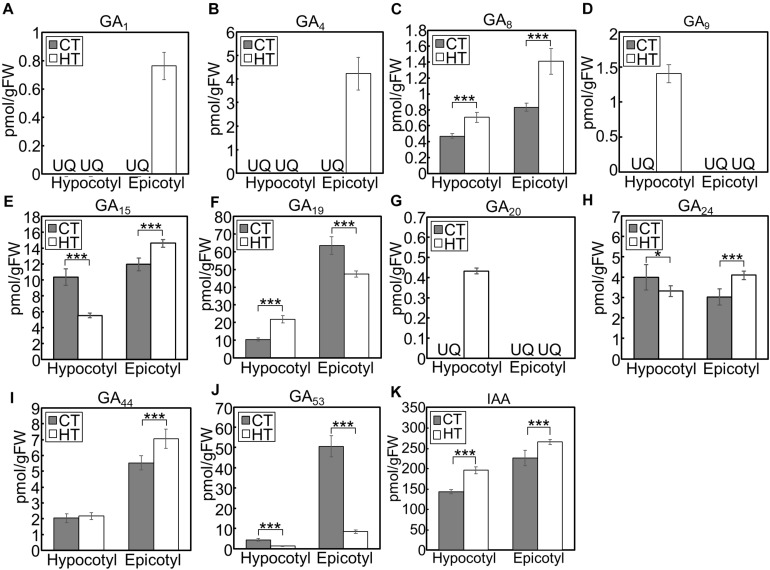
GA and IAA concentration in hypocotyls and epicotyls under two different temperatures. Hypocotyls and epicotyls of tomato seedlings grown for 7 days under control and high temperatures were harvested and subjected to hormone analysis. The concentrations of GAs **(A–J)** and IAA **(K)** were quantified using liquid chromatography-tandem mass spectrometry. Data are represented as mean ± SD (*n* = 4 or 5). *P < 0.1; ***P < 0.01 (Student’s *t* test). GA_7_ and GA_12_ were below the quantification limit. CT, control temperature; HT, high temperature; FW, fresh weight; UQ, under quantification limit.

When we analyzed hormone species under the CT and HT positive DIF treatments (Figure 7), GA_1_ and GA_4_ were only detected in epicotyls under the HT treatment (Figures 7A,B). Concentrations of the inactive precursors GA_15_, GA_24_, and GA_44_ in epicotyls were higher under the HT treatment than those under the CT treatment (Figures 7E,H,I). Conversely, concentrations of GA_53_ were lower under the HT treatment those under the CT treatment (Figure 7J). These patterns were opposite to the changes that occurred following negative DIF treatment (Figure 6).

IAA concentrations in negative DIF-treated hypocotyls and epicotyls were slightly but significantly lower than those in positive DIF-treated tissues (Figure 6K). In contrast, the concentrations of IAA in hypocotyls and epicotyls were higher under HT than those under CT (Figure 7K). These results were consistent with the expression pattern of IAA biosynthesis and signaling genes (Figures 4, 5).

We also quantified the concentration of cytokinins (CKs) because the involvement of this hormone in vascular development is well-documented (Kieber and Schaller, 2014). Several *N*^6^-(Δ^2^-isopentenyl) adenine (iP)-type and *trans*-zeatin-type (tZ-type) species had higher concentrations in negative DIF-treated epicotyls than positive DIF-treated epicotyls (Supplementary Figure 4). Conversely, an opposite effect was found under the CT and HT treatments (Supplementary Figure 5).

## Discussion

In this study, we demonstrated that DT and NT affect stem growth in tomato seedlings, and that this is possibly mediated by the regulation of GA-, IAA-, and cell wall-related genes. Under the positive DIF treatment, higher temperatures promoted stem elongation, and this was accompanied by the upregulation of GA and IAA synthesis genes, resulting in higher concentrations of their active forms. Conversely, negative DIF inhibited stem elongation, which was accompanied by the downregulation of GA and IAA synthesis genes and decrease in GA and IAA concentrations. Although numerous studies have investigated plant growth regulation in response to temperature, our study provides information about possible mechanisms regulating stem growth under DIF treatments.

Under the negative DIF treatment, the concentrations of GA_1_, GA_4_, and some GA precursors were lower, whereas those of GA_53_ were higher, compared with those under positive DIF treatment. Furthermore, the expression of *SlGA20ox1* was downregulated under the negative DIF treatment. In contrast, under the positive DIF treatment (i.e., higher temperatures), upregulation of *SlGA20ox1* was observed and this treatment had the opposite effect on the profile of GA_1_, GA_4_ and the precursors, compared to that of the negative DIF treatment. Since *GA20ox* catalyzes the following multi-step reactions: GA_12_/GA_53_→GA_15_/GA_44_→GA_24_/GA_19_→GA_9_/GA_20_, these results were consistent with the downregulation or upregulation of *SlGA20ox1* expression. In addition, as GA_8_ is the inactivated form of GA_1_, lower GA_8_ concentrations in negative DIF-treated epicotyls supported weakened GA activity. *GA20ox* plays a key role in the GA biosynthesis pathway and affects bioactive GA content (Yamaguchi, 2008). In *A. thaliana*, the over-expression of *GA20ox* enhances hypocotyl elongation (Huang et al., 1998; Coles et al., 1999; Ferrero et al., 2019), and the expression of *GA20ox* (*GA20ox1*) is upregulated by high temperatures in hypocotyls (Stavang et al., 2009). Thus, it is suggested that *SlGA20ox1* plays a key role in regulating stem elongation in tomato seedlings in response to different DIF conditions.

IAA is involved in thermomorphogenesis, such as stem elongation, in response to higher temperatures (Quint et al., 2016). In *A. thaliana* hypocotyls, high temperatures induce the expression of *YUC*, *TAA* and *SAUR*, which facilitates stem elongation (Stavang et al., 2009; Franklin et al., 2011). In our analysis, these homologs were downregulated under the negative DIF treatment and upregulated under the high temperature positive DIF treatment, suggesting that these IAA-related genes also play a role in regulating stem elongation in tomato in response to temperature conditions.

Expression patterns of the cell wall-related genes *SlEXP1*, *SlEXP2*, and *SlXTH2* were correlated with GA- and IAA-biosynthesis genes. EXPs are cell wall loosening proteins, which cause cell wall expansion (Marowa et al., 2016). Previous studies have shown that *EXPs* are involved in stem elongation of *Oryza sativa* (Cho and Kende, 1997a,b; Lee and Kende, 2001, 2002; Choi et al., 2003; Zou et al., 2015) and respond to GA (Cho and Kende, 1997b; Lee and Kende, 2001, 2002). *EXP1* has been found to be regulated by temperature in *Agrostis scabra* and *Agrostis stolonifera* (Xu et al., 2007). XTH catalyzes Xyloglucan endohydrolysis and endotransglycosylation, which is involved in the modification of cell wall structures (Rose et al., 2002). It has also been reported that *EXPs* and *XTHs* are regulated by IAA (Goda et al., 2004; Majda and Robert, 2018; Lehman and Sanguinet, 2019). The response of cell wall-related genes to temperature might be mediated by GA and/or IAA action in tomato seedlings.

Previous studies investigating the molecular mechanisms behind temperature acclimation in *A. thaliana* identified PIF4 as a key regulator (Proveniers and van Zanten, 2013; de Wit et al., 2014; Quint et al., 2016). Expression of *PIF4* was upregulated by high temperatures and was found to control GA and IAA biosynthesis and signaling (Koini et al., 2009; Stavang et al., 2009; Franklin et al., 2011; Sun et al., 2012). In our experiment, *SlPIF4* was upregulated in epicotyls under the high temperature positive DIF treatment, suggesting that similar regulatory systems are employed in tomato seedlings. Conversely, *SlPIF4* expression was not affected by the negative DIF treatment. The role of PIF4 in the regulation of stem elongation under negative DIF remains to be clarified. Future studies should focus on constructing a loss-of-function mutant to help understand its role.

Stem thickness and vessel development did not differ significantly between the negative and positive DIF treatments. Limited studies have reported the effect of temperature on stem thickness and vessel development. Among the phytohormones, CKs play an important role in vascular development (Kieber and Schaller, 2014). In a cytokinin biosynthesis mutant of *A. thaliana*, stem thickness and the number of xylems were significantly decreased (Matsumoto-Kitano et al., 2008). Another recent study suggests that CK is involved in the variation in xylem development in Dutch and Japanese tomato cultivars (Qi et al., 2020). However, in our experimental conditions, the concentration of endogenous cytokinin was increased in the negative DIF and decreased in the positive DIF treatments, suggesting that CK plays a minor role in the regulation of xylem development under these DIF treatments. Further analysis is needed to elucidate the mechanisms underlying temperature-dependent regulation of stem thickness and xylem development.

It is believed that the quality of seedlings greatly affects the final agricultural yield. Our results show that short-term negative DIF treatment can control plant height without affecting stem thickness. This might be an effective technique for growing tomatoes in nurseries. Since stem thickening is accompanied by vascular development, it may give a positive effect on mineral transport, the partition of assimilates, and fruit growth. Findings in this study would shed light on mechanisms of possible new agricultural practices.

## Materials and Methods

### Plant Materials and Growth Conditions

The tomato (*S*. *lycopersicum*) cultivar used in this study was Managua RZ (RIJK ZWAAN, Netherlands), except for the growth comparison of tomato cultivars under DIF treatments (Supplementary Figure 3). In the comparison experiment, we used CF Momotaro-York and Daiki B Baria (Takii Seed, Kyoto, Japan), and Rinka and Reiyo (Sakata Seed, Kanagawa, Japan).

Tomato seeds were imbibed in water in a petri dish at 28°C in the dark for 2 d. After imbibition, germinated seeds were transferred to a wet rockwool block (Nippon Rockwool Corporation, Japan) and were grown at 23°C/23°C (DT/NT, 16 h photoperiod) under 130 to 140 μmol photons m^–2^ s^–1^ of fluorescent illumination for 5–6 d. Next, the young seedlings, whose cotyledons were fully opened, were further grown on the rockwool block with liquid culture medium under the following two temperature-condition pairs: 25°C/20°C and 30°C/25°C, or 30°C/25°C and 25°C/30°C (DT/NT, 16 h photoperiod) under 300 μmol photons m^–2^ s^–1^ of light for 7 d (Supplementary Figure S1). The liquid culture medium contained nutrients at the following concentrations: 5 mM KNO_3_, 1 mM NH_4_H_2_PO_4_, 0.5 mM MgSO_4_, 5.5 mM Ca(NO_3_)_2_, 27 μM Fe-EDTA, 25 μM KCl, 10 μM H_3_BO_3_, 1 μM MnSO_4_, 1 μM ZnSO_4_, 0.25 μM CuSO_4_, and 0.04 μM Na_2_MoO_4_.

### Fluorescent Observation of Xylem

The middle section of the tomato seedling hypocotyls was harvested and fixed in a 4% paraformaldehyde phosphate buffer solution (Nacalai Tesque, Kyoto, Japan). The fixed hypocotyls were thinly sliced (∼0.5 mm) with a razor blade, and cross sections were observed with fluorescence microscopy (Mirror unit with U-FUW, Olympus BX53, Olympus, Japan). The diameter of vessels was measured using the ImageJ software (Abramoff et al., 2004).

### GA, IAA, and CK Quantification

Phytohormones were extracted and semi-purified as previously described (Kojima et al., 2009; Kojima and Sakakibara, 2012). CKs were quantified using an ultra-performance liquid chromatography (UPLC)-electrospray interface (ESI) tandem quadrupole mass spectrometer (qMS/MS) (AQUITY UPLC^TM^ System/Xevo-TQS; Waters, Milford, MA, United States) as described previously (Kojima et al., 2009) with an ODS column (AQUITY UPLC HSS T3, 1.8mm, 2.1 x 100 mm; Waters). IAA and GAs were quantified using an ultra-high performance liquid chromatography (UHPLC)-ESI quadrupole-orbitrap mass spectrometer (UHPLC/Q-Exactive^TM^; Thermo Fisher Scientific, United States) as described previously (Kojima and Sakakibara, 2012; Shinozaki et al., 2015) with an ODS column (AQUITY UPLC HSS T3, 1.8mm, 2.1 x 100 mm; Waters).

### RNA Extraction

Hypocotyls and epicotyls were frozen in liquid nitrogen and ground to a fine powder with a mortar and pestle. Total RNA was extracted using an RNeasy Mini kit with an RNase-Free DNase Set (Cat. No. 74104/79254, Qiagen, Hilden, Germany). The RNA in samples was quantified using a NanoDrop-1000 spectrophotometer and the quality was monitored using an Agilent 2100 Bioanalyzer (Agilent Technologies, Santa Clara, CA, United States).

### Microarray and Data Analysis

Total RNA was extracted from epicotyls of young seedlings grown for 7 d under 30°C/25°C (positive DIF) and 25°C/30°C (negative DIF) (DT/NT, 16 h photoperiod) as described in the preceding subsection. Three biological replicates were used for microarray analysis. Target labeling was performed according to the manual of the Low Input Quick Amp Labeling Kit, One-Color (Agilent Technologies). We used a tomato custom-designed microarray (platform ID “GPL21511”). Hybridization was performed according to the manufacturer’s instructions. We scanned the microarray images using an Agilent DNA Microarray Scanner G2565CA (Agilent Technologies). Scanned images were converted to signal data using Feature Extraction software (Agilent Technologies). Value definition in the data matrix was Log2. GO enrichment analysis among DEGs (top 300 upregulated and top 300 downregulated genes; *P* < 0.05) was performed using the GO Analysis Toolkit and Database for Agricultural Community (AgriGO, http://systemsbiology.cau.edu.cn/agriGOv2/) (Tian et al., 2017).

### Reverse Transcriptase Real-time PCR

First-strand cDNA was synthesized using SuperScript^TM^ III First-Strand Synthesis SuperMix (Invitrogen, Waltham, MA, United States). Real-time PCR was performed using a StepOnePlus Real Time PCR system (Applied Biosystems, Waltham, MA, United States) and a KAPA SYBR FAST qPCR Master Mix (2×) Kit (Kapa Biosystems, London, United Kingdom) under the following conditions: 95°C for 3 min; followed by 40 cycles of 95°C for 3 s and 60°C for 20 s. Gene expression was calculated using the ΔΔCT method and normalized to that of the ubiquitin homolog as the housekeeping gene (Solyc01g056940). The following primers were used: for the housekeeping gene (Solyc01g056940), forward primer 5′-CG TGGTGGTGCTAAGAAGAG-3′, reverse primer 5′-ACGAAG CCTCTGAACCTTTC-3′; for *SlGA20ox1* (Solyc03g006880), forward primer 5′-TGGCGTTCCATCAGTCCAAA-3′, reverse primer 5′-TTCGAGGGTTGTTGGAGTCC-3′; for *SlTAA1* (Solyc05g031600), forward primer 5′-TGAAGCACACCCTGC ATTTG-3′, reverse primer 5′-ACTTCCAAATCTTCTTCCACT CCTT-3′; for *SlYUC* (Solyc08g068160), forward primer 5′-GC CCTCGTGGCTAAAGGAA-3′, reverse primer 5′-CCACTGCA TAAAGTCCACACTCTC-3′; for *SlSAUR47* (Solyc04g053000), forward primer 5′-GAAGAACAGTTTGGCTTCGATTAC-3′, reverse primer 5′-CGGTATGTGAGATCAACAAACA-3′; for *SlEXP2* (Solyc06g049050), forward primer 5′-TTCGAAGGGTG CCCTGTAT-3′, reverse primer 5′-TGAATATCACCAGCAC CTCCA-3′; for *SlEXP1* (Solyc06g051800), forward primer 5′-CGCTGGCATTGTTCCTGT-3′, reverse primer 5′-CTGC ACCTGCTACATTCGTG-3′; for *SlXTH2* (Solyc07g009380), forward primer 5′-TATGCACAAGGCAAGGGAGA-3′, reverse primer 5′-TGTATTGTCTTATTGGTGTCCCATC-3′; for *SlPIF4* (Solyc07g043580), forward primer 5′-ATCAAGCAGCTGCAAT GTGC-3′, reverse primer 5′-CTGCTGAGTTTGCTGTGCTG-3′.

## Data Availability Statement

Microarray data have been deposited in the National Center for Biotechnology Information Gene Expression Omnibus (NCBI GEO) database under accession number GSE131496.

## Author Contributions

KO, AY, SI, and HS designed research. KO, AY, YK, KF, MK, YT, and KY performed research. KO and YK analyzed the data. KO and HS wrote the manuscript. All authors contributed to the article and approved the submitted version.

## Conflict of Interest

The authors declare that the research was conducted in the absence of any commercial or financial relationships that could be construed as a potential conflict of interest.

## Supplementary Material

Supplementary Figure 1 Schematic representation of growth conditions in the study. **(A)** Control temperature treatment, 25°C/20°C (DT/NT). **(B)** High temperature/positive DIF treatment, 30°C/25°C (DT/NT). **(C)** Negative DIF treatment, 25°C/30°C (DT/NT). Young tomato seedlings were grown for 7 days under each condition. White bars show the light period, black bars show the dark period, and red arrowheads show the sampling time for the experiments. Further details are explained in the Materials and Methods.

Supplementary Figure 2 Growth of tomato plants under negative and positive DIF treatments. Young tomato seedlings were grown for 7 days under positive and negative DIF treatments. **(A)** Growth of seedlings. Seedlings of tomato were photographed at 0, 1, 3, 5, and 7 days. Scale bars = 5 cm. (**B,C**) Hypocotyl and epicotyl length, respectively. (**D,E**) Hypocotyl and epicotyl diameters, respectively. **(F)** Cotyledons and true leaves photographed at 7 days after onset of DIF treatments. Scale bars = 5 cm. Data are represented as mean ± SD (*n* = 3 or 4). ^∗^*P* < 0.05; ^∗∗^*P* < 0.01 (Student’s *t* test). +DIF, positive DIF; -DIF, negative DIF.

Supplementary Figure 3 Growth comparison of five tomato cultivars under DIF treatments. Seedlings of five tomato cultivars, Daiki B baria (DaikiB), Rinka, CF momotaro-york (CFMY), Managua RZ (MA), and Reiyo (RYO) were grown for 7 days under positive and negative DIF. **(A)** Growth of seedlings. Seedlings of tomato were photographed at 7 days. Scale bars = 5 cm. (**B,C**) Hypocotyl and epicotyl length, respectively. (**D,E**) Hypocotyl and epicotyl diameter, respectively. Data are shown as boxplots (*n* = 3). Cross marks in boxplot indicate the mean values. ^∗^*P* < 0.05; ^∗∗^*P* < 0.01 (Student’s *t* test). +DIF, positive DIF; -DIF, negative DIF.

Supplementary Figure 4 Concentration of CKs in hypocotyls and epicotyls under positive and negative DIF treatments. Hypocotyls and epicotyls of tomato seedlings grown for 7 days under positive and negative DIF treatments were harvested and subjected to hormone analysis. The concentration of CKs were quantified using liquid chromatography-tandem mass spectrometry. Data are represented as mean ± SD (*n* = 3 to 5). tZ, *trans*-zeatin; tZR, tZ riboside; tZRPs, tZR 5′-phosphates; tZ7G, tZ-*7N*-glucoside; tZ9G, tZ-*9N*-glucoside; tZOG, tZ-*O*-glucoside; tZROG, tZR-*O*-glucoside; iP, *N*^6^-(*Δ^2^*-isopentenyl)adenine; iPR, iP riboside; iPRPs, iPR 5′-phosphates; iP7G, iP-*7N*-glucoside; iP9G, iP-*9N*-glucoside. ^∗^*P* < 0.1; ^∗∗^*P* < 0.05; ^∗∗∗^*P* < 0.01 (Student’s *t* test). tZRPOG, tZRP-*O*-glucoside was below the quantification limit. +DIF, positive DIF; -DIF, negative DIF; FW, fresh weight; UQ, under quantification limit.

Supplementary Figure 5 Concentration of CKs in hypocotyls and epicotyls under two different temperatures. Hypocotyls and epicotyls of tomato seedlings grown for 7 days under control and high temperatures were harvested and subjected to hormone analysis. The concentration of CKs were quantified using liquid chromatography-tandem mass spectrometry. Data are represented as mean ± SD (*n* = 3 to 5). tZ, *trans*-zeatin; tZR, tZ riboside; tZRPs, tZR 5′-phosphates; tZ7G, tZ-*7N*-glucoside; tZ9G, tZ-*9N*-glucoside; tZOG, tZ-*O*-glucoside; tZROG, tZR-*O*-glucoside; iP, *N*^6^-(*Δ^2^*-isopentenyl)adenine; iPR, iP riboside; iPRPs, iPR 5′-phosphates; iP7G, iP-*7N*-glucoside; iP9G, iP-*9N*-glucoside. ^∗^*P* < 0.1; ^∗∗^*P* < 0.05; ^∗∗∗^*P* < 0.01 (Student’s *t* test). tZRPOG, tZRP-*O*-glucoside was below the quantification limit. CT, control temperature; HT, high temperature; FW, fresh weight; UQ, under quantification limit.

## Abbreviations

CT: control temperature
DT: day temperature
+DIF: positive DIF
-DIF: negative DIF
HT: high temperature
NT: night temperature.
