# Supplementary material for: Difference Between Day and Night Temperatures Affects Stem Elongation in Tomato (Solanum lycopersicum) Seedlings via Regulation of Gibberellin and Auxin Synthesis
Source: Front Plant Sci. 2020 Dec 8;11:577235. doi: 10.3389/fpls.2020.577235 (PMC7752778; doi:10.3389/fpls.2020.577235)
Supplement: Supplementary Figure 1 — Schematic representation of growth conditions in the study. (A) Control temperature treatment, 25°C/20°C (DT/NT). (B) High temperature/positive DIF treatment, 30°C/25°C (DT/NT). (C) Negative DIF treatment, 25°C/30°C (DT/NT). Young tomato seedlings were grown for 7 days under each condition. White bars show the light period, black bars show the dark period, and red arrowheads show the sampling time for the experiments. Further details are explained in the Materials and Methods. [file Presentation_1.PDF]

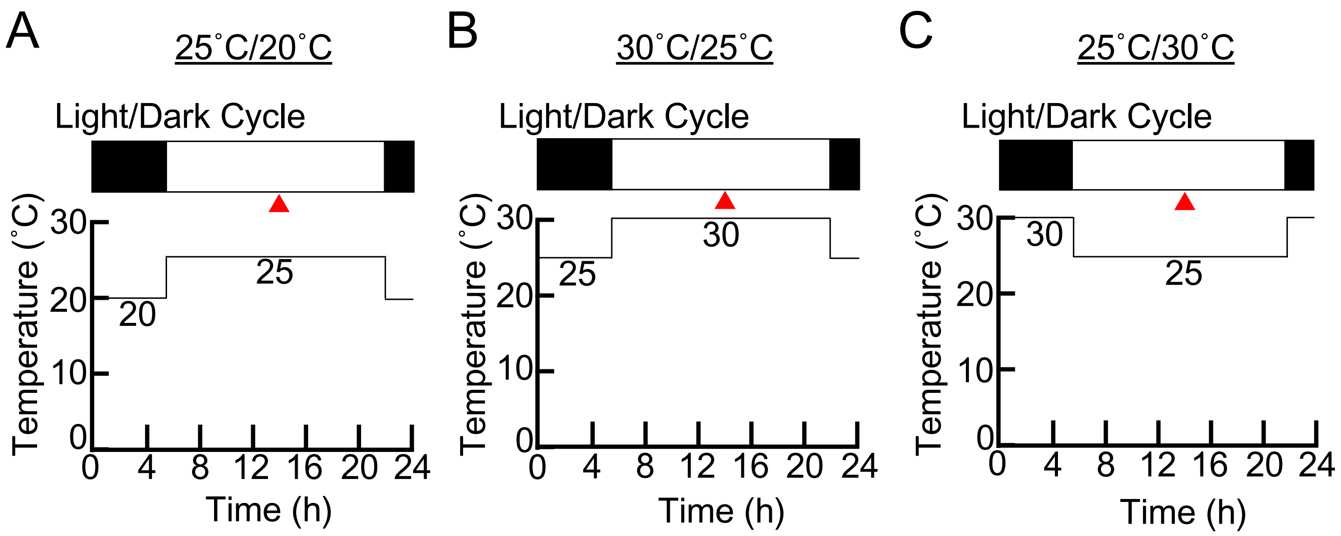

Supplemental Figure S1.

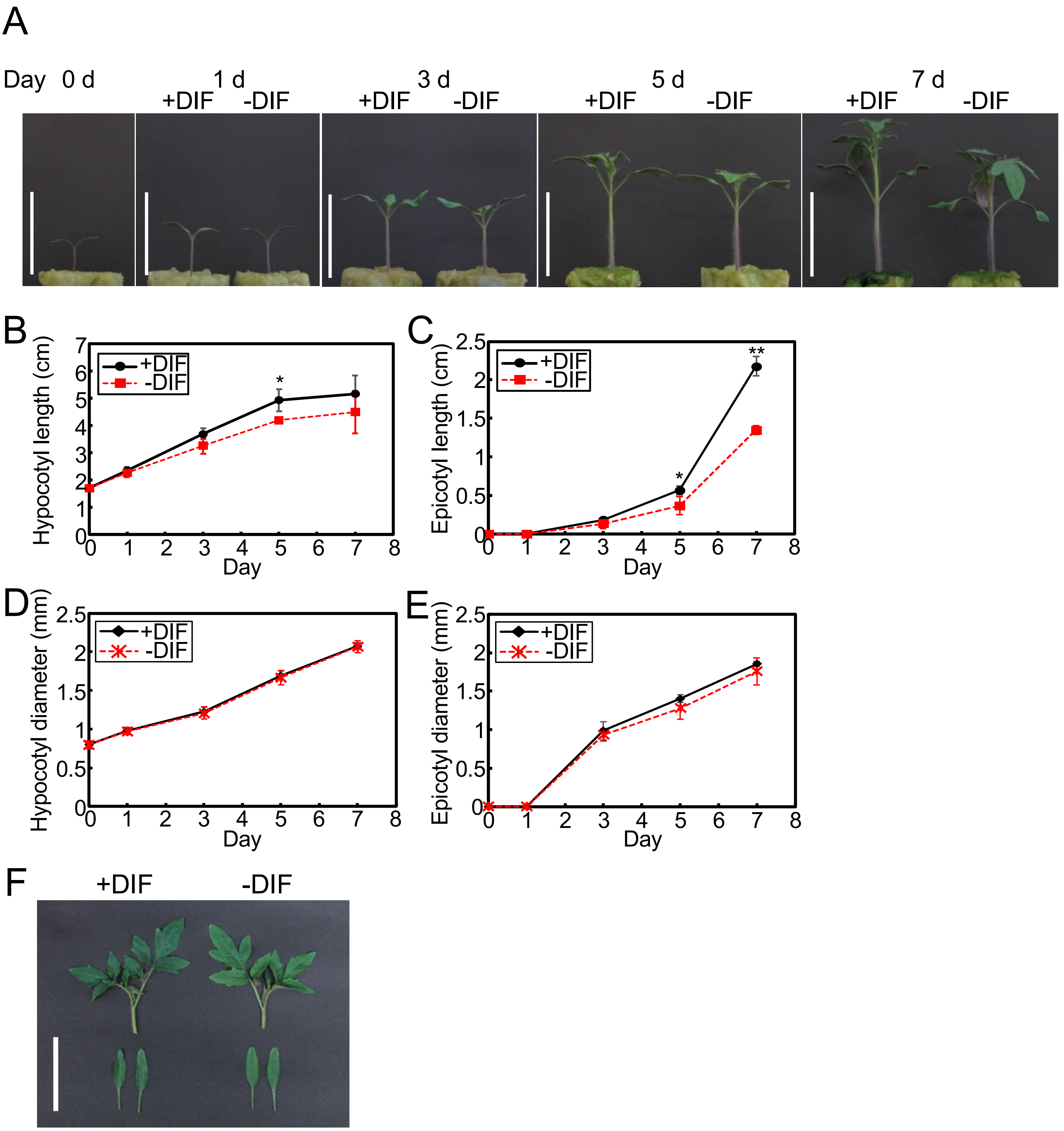

Supplemental Figure S2.

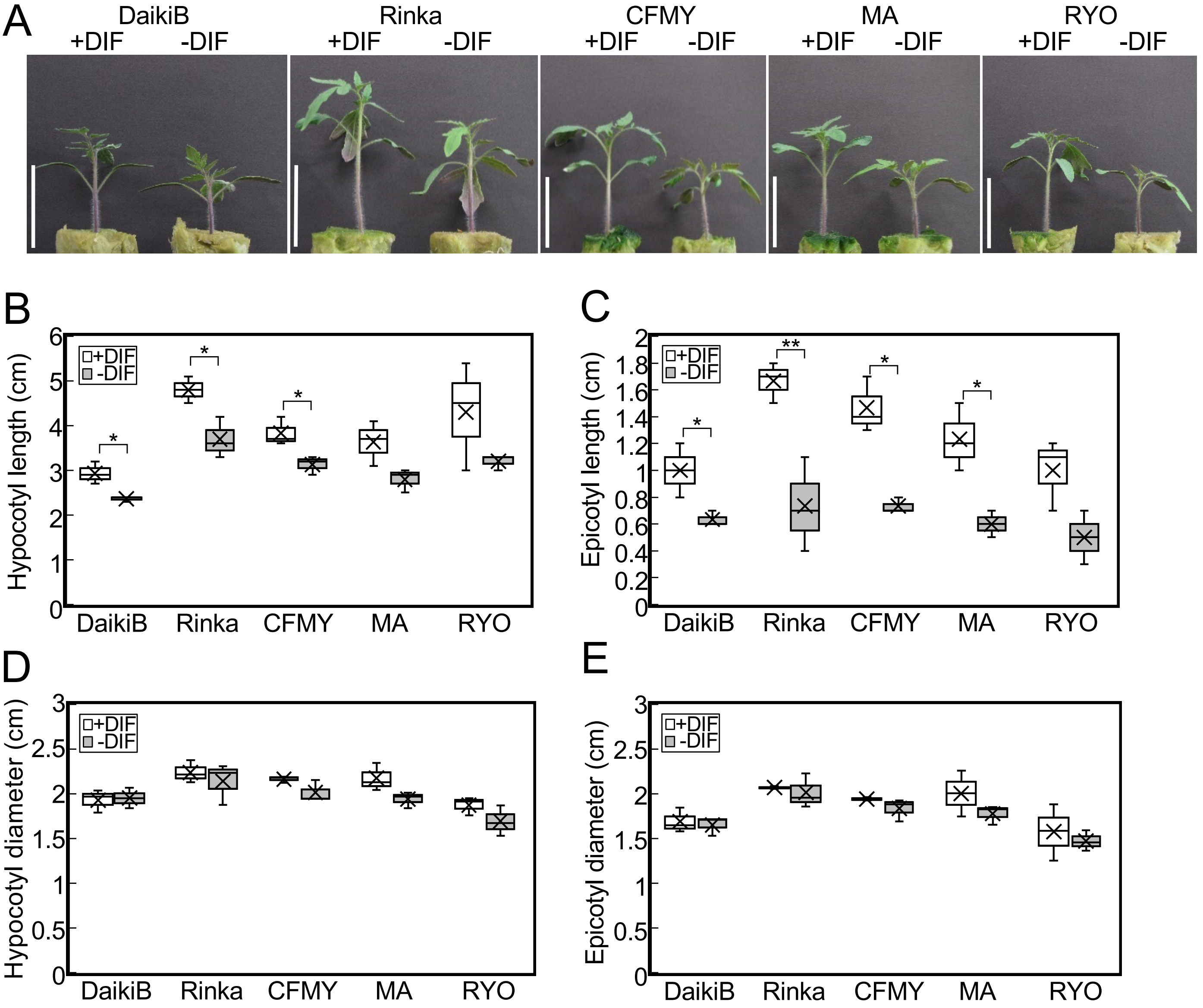

Supplemental Figure S3.

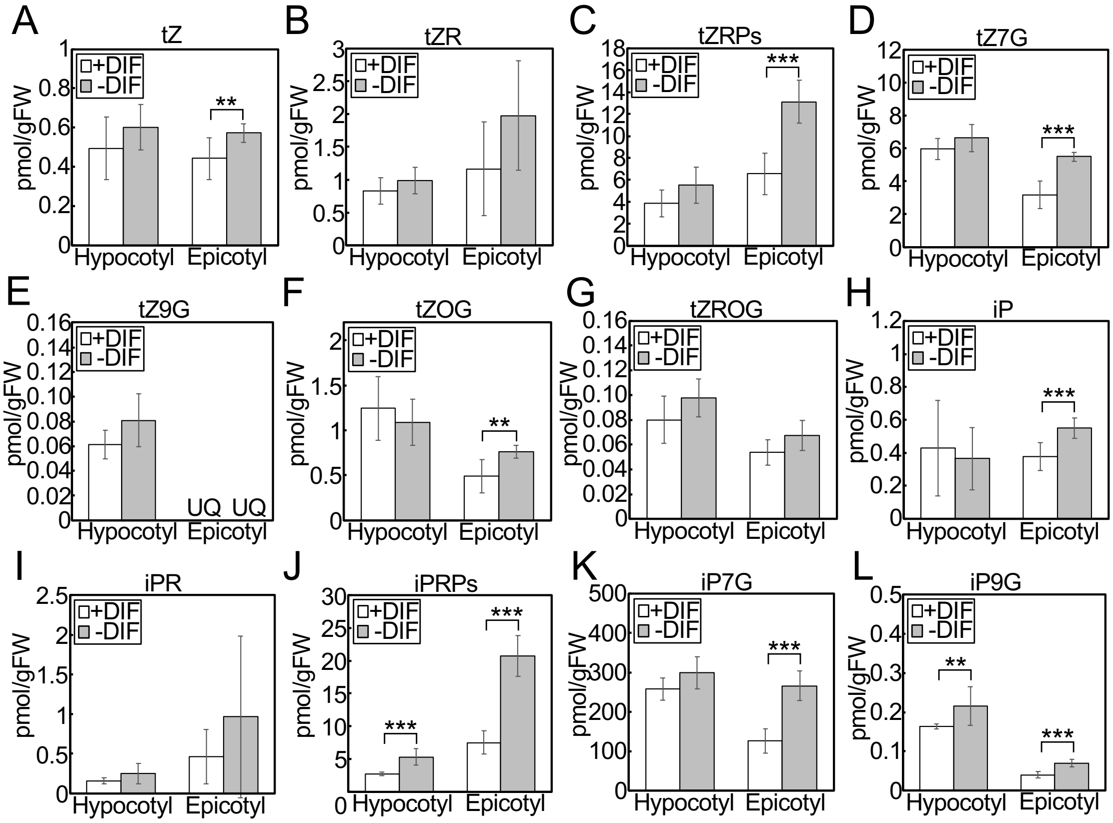

Supplemental Figure S4.

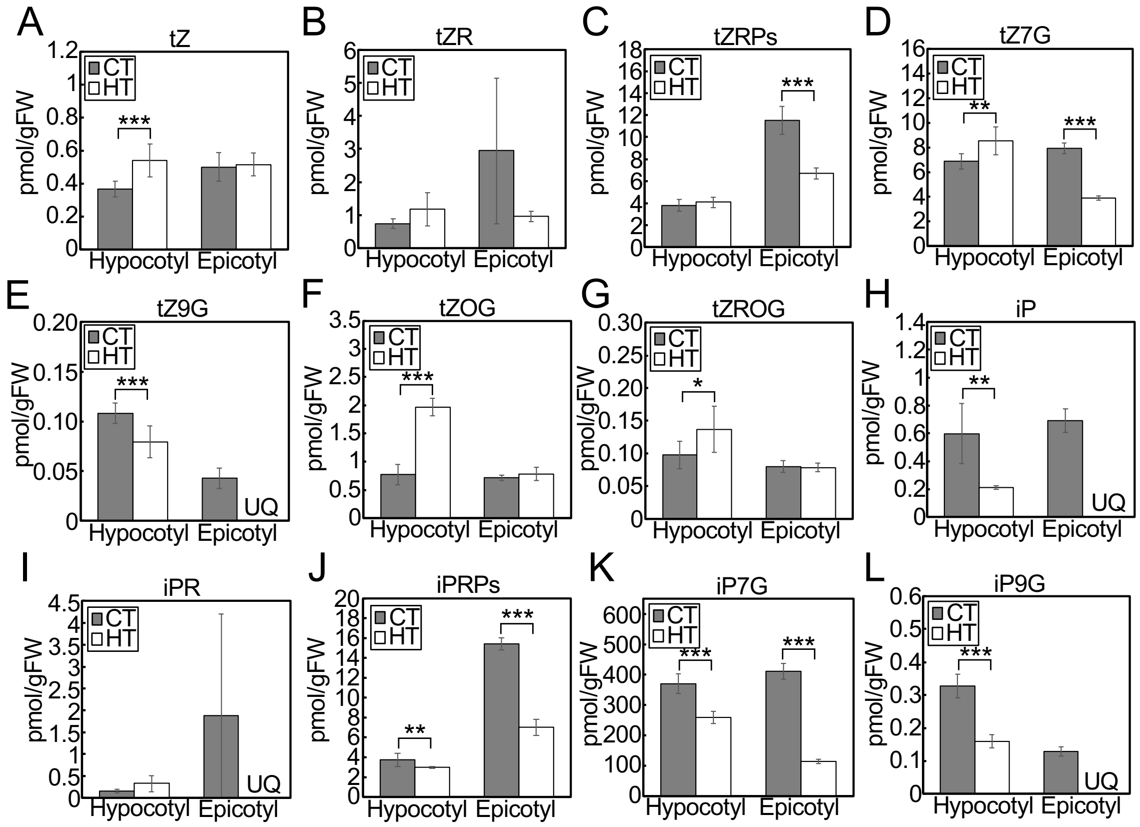

Supplemental Figure S5.
